# Development and validation of an explainable neural network model for predicting progression in type 2 diabetic kidney disease

**DOI:** 10.3389/fendo.2026.1858808

**Published:** 2026-05-28

**Authors:** Binfeng Xiong, Chengzheng Duan, Keying Lin, Sheng Xu

**Affiliations:** 1 Jin hua Graduate Joint Training Base, Zhejiang Chinese Medical University, Hangzhou, China; 2 The Quzhou Affiliated Hospital of Wenzhou Medical University, Quzhou People’s Hospital, Quzhou, China; 3 Department of Nephrology, Quzhou Hospital of Traditional Chinese Medicine, Quzhou, China

**Keywords:** machine learning, neural network, predictive model, serum creatinine, type 2 diabetic kidney disease, urinary albumin-to-creatinine ratio

## Abstract

**Background:**

Type 2 diabetic kidney disease (T2DKD) affects 20–40% of patients with type 2 diabetes mellitus and has become the leading cause of end-stage renal disease globally. Early identification of patients at risk of rapid progression remains challenging, as existing prediction models often rely on complex indicators unsuitable for primary care settings. This study aimed to develop and validate a machine learning model using routine clinical parameters to predict T2DKD progression.

**Methods:**

This single-center retrospective cohort study enrolled 349 patients diagnosed with T2DKD according to clinical criteria at Quzhou People’s Hospital in China between June 2022 and June 2025. From 36 baseline characteristics, four core predictors were identified through least absolute shrinkage and selection operator (LASSO) regression and multivariable logistic regression. Six machine learning models were constructed, and model performance was evaluated by discrimination, calibration, and clinical net benefit. Model interpretability was assessed using SHapley Additive exPlanations (SHAP) analysis.

**Results:**

Metabolic dysfunction-associated steatotic liver disease (MASLD), aspartate aminotransferase (AST), diabetic peripheral neuropathy (DPN), and age were identified as independent core predictors. The neural network (NN) model achieved optimal performance in the test dataset, with an area under the curve (AUC) of 0.742, satisfactory calibration (Hosmer–Lemeshow P = 0.2020), the lowest Brier score (0.2105), and superior clinical net benefit across risk thresholds of 0.2–0.6. SHAP analysis confirmed stable feature importance rankings between training and test datasets (Pearson r = 0.976) and revealed synergistic interactions among MASLD, AST, and DPN.

**Conclusion:**

A NN model incorporating four routine clinical indicators effectively predicts T2DKD progression risk. This cost-effective tool is suitable for clinical practice and community health services, offering a scalable solution for early intervention and prognosis improvement.

## 1 Introduction

Type 2 Diabetic Kidney Disease (T2DKD) is one of the most devastating microvascular complications of type 2 diabetes mellitus (T2DM). It affects approximately 20% to 40% of patients with T2DM worldwide (1, 2) and has become the leading cause of end-stage renal disease (ESRD) globally. According to the Global Burden of Disease (GBD) 2021 study, the global number of patients with DKD increased by approximately 74% from 14 million cases in 1990 to 24 million cases in 2021 (3), of which T2DKD accounts for more than 80%. China has become one of the countries with the largest number of patients living with T2DKD worldwide. Meanwhile, the continuous growth in the number of T2DKD patients has imposed an increasingly heavy economic burden on healthcare systems around the world (4). For example, the total annual treatment cost for a single patient with T2DKD in the United States is as high as 25,000 US dollars (5). Therefore, early identification of T2DKD patients at risk of rapid disease progression is currently a key research priority and an urgent clinical need in this field.

Proteinuria is one of the core early hallmarks of DKD. Its pathological essence is the increased permeability of the glomerular filtration membrane, which leads to the loss of protein through urine. The urinary albumin-to-creatinine ratio (UACR), a non-invasive assay, is routinely used in clinical practice to evaluate the severity of proteinuria. Proteinuria level is an important indicator for the prognostic assessment of DKD, yet it has limited predictive efficacy for the risk of ESRD onset, with the drawback of insufficient specificity and sensitivity (6). Serum creatinine (Scr) is a core biomarker reflecting advanced renal function impairment (7). In clinical settings, UACR and Scr are often combined to comprehensively assess the disease progression status of T2DKD.

Traditional clinical prediction models have notable limitations in clinical application, as they are difficult to capture the complex non-linear relationships and interactions among multi-dimensional risk factors. Machine learning (ML), an interdisciplinary data analysis technology, can mine deep-seated patterns in real-world clinical data through various algorithms (8). In recent years, ML algorithms have been widely used in the medical field, covering the early prevention and diagnosis of diseases (9), new drug research and development (10), and the prevention and control of infectious diseases (11).

Current predictive studies using ML in the DKD field mostly focus on predicting the incidence risk of DKD in patients with T2DM (12–14). These studies included DM populations without a confirmed diagnosis of DKD as research subjects, rather than patients with established DKD. There is a relative paucity of predictive studies on disease progression in patients with confirmed T2DKD. Meanwhile, some existing prediction models incorporate numerous and complicated indicators with high detection costs, which are difficult to popularize and apply in primary healthcare institutions (15, 16). On this basis, we included patients’ routinely available demographic data and laboratory parameters in this study. We then developed and compared the predictive performance of six ML models for T2DKD progression, identified the optimal prediction model, and validated its clinical application value.

## 2 Materials and methods

### 2.1 Data sources and participant selection

This was a single-center retrospective cohort study conducted at Quzhou People’s Hospital, Zhejiang, China. Consecutive patients with T2DKD who visited the outpatient clinics or were admitted to the inpatient departments between June 2022 and June 2025 were enrolled. All diagnoses of T2DKD followed the clinical criteria specified in the 2025 Asia Pacific Society of Nephrology (APSN) Clinical Practice Guideline for Diabetic Kidney Disease (17).

The study was performed in strict accordance with the Declaration of Helsinki. The protocol was approved by the Ethics Committee of Quzhou People’s Hospital (Approval No.: MR-33-25-082067). The requirement for written informed consent from participants was waived by the ethics committee due to the retrospective nature of the study.

### 2.2 Inclusion criteria and exclusion criteria

Inclusion criteria were as follows (1): diagnosis of T2DKD according to the 2025 APSN guideline (17); (2) age ≥18 years; and (3) absence of severe comorbidities that may impair renal function or urinary protein metabolism, including severe cardiovascular or cerebrovascular disease, end-stage liver disease, uncontrolled autoimmune disorders, and severe hematological diseases.

Exclusion criteria were as follows: (1) absence of T2DKD; (2) acute kidney injury; (3) active infection or bleeding diathesis; (4) other renal diseases associated with proteinuria, such as urinary tract infection, acute or chronic glomerulonephritis, nephrotic syndrome, or lupus nephritis; (5) histologically confirmed malignancy or history of cancer treatment within the preceding 5 years; (6) pregnancy or lactation; and (7) incomplete clinical data that could bias outcome evaluation.

### 2.3 Sample size calculation

Sample size was calculated using PASS 15.0 software based on the Events Per Variable (EPV) method. Statistical standards for clinical prediction models require EPV > 20 for ML models (18). This study included 4 core predictive variables and used a 20-fold EPV threshold. The estimated event rate was 0.45, and the training datasets accounted for 70% of the total sample. The minimum required sample size for the training datasets was 177.8 cases. After accounting for 10% invalid samples, the required total sample size was at least 283 cases. In practice, 349 patients were enrolled, with 158 cases of disease progression. The final model EPV was 27.5, which fully satisfied the requirements for model construction and internal validation.

### 2.4 Participant grouping and outcome definition

Baseline data were collected at the time of initial T2DKD diagnosis. Follow-up data were collected within a 3-year window after diagnosis. Participants were stratified into progression and non-progression groups based on changes in UACR or Scr from baseline to the last follow-up visit, with progression defined by meeting any of the following criteria: (1) progression from A1 to A2 stage; (2) progression from A2 to A3 stage; (3) a ≥2-fold increase in UACR from baseline in patients with A2-stage disease without reaching A3 thresholds; or (4) a ≥2-fold increase in Scr from baseline in patients with A3-stage disease.

Renal injury was staged using the albuminuria categories from the 2024 KDIGO Clinical Practice Guideline for the Evaluation and Management of Chronic Kidney Disease (19). Staging criteria were defined as follows: (1) A1 stage, UACR <30 mg/g; (2) A2 stage, 30 mg/g ≤ UACR <300 mg/g; and (3) A3 stage, UACR ≥300 mg/g.

### 2.5 Data collection

All data were extracted from electronic health records in the Hospital Information System between June 2022 and June 2025. T2DKD diagnosis followed the 2025 Asia Pacific Society of Nephrology (APSN) Guideline (17). Renal injury staging and outcome determination followed the 2024 KDIGO Guideline (19). Final diagnoses were confirmed by review of baseline and last follow-up assessments. Three hundred forty-nine eligible patients were enrolled.

Collected variables were categorized into four groups: (1) demographic characteristics: age, sex, body mass index (BMI), smoking status, and drinking status; (2) medical history and comorbidities: duration of diabetes mellitus (DM duration), duration of hypertension (HTN duration), diabetic retinopathy (DR), diabetic peripheral neuropathy (DPN), osteoporosis (OP), and metabolic dysfunction-associated steatotic liver disease (MASLD); (3) medication history: metformin (Met) use and sodium-glucose cotransporter 2 inhibitor (SGLT2i) use; and (4) physical examination and laboratory parameters: systolic blood pressure (SBP), diastolic blood pressure (DBP), fasting plasma glucose (FPG), vitamin D (VD), parathyroid hormone (PTH), glycated hemoglobin A1c (HbA1c), hemoglobin (HGB), white blood cell count (WBC), platelet count (PLT), alanine aminotransferase (ALT), aspartate aminotransferase (AST), aspartate-to-alanine aminotransferase ratio (AAR), alkaline phosphatase (ALP), total bilirubin (TBIL), direct bilirubin (DBIL), albumin (ALB), total cholesterol (CHOL), high-sensitivity C-reactive protein (hs-CRP), low-density lipoprotein (LDL), triglyceride (TG), high-density lipoprotein (HDL), blood urea nitrogen (BUN), and uric acid (UA).

### 2.6 Statistical analysis and experimental design

All statistical analyses were performed using R software (version 4.4.2). Detailed package names, versions, random seeds for reproducibility, and the complete data splitting protocol are provided in **Supplementary Materials** (**Section S2**). Missing data patterns were first examined. Missing data for baseline characteristics were imputed using the random forest imputation method. The proportion and pattern of missingness for each variable, as well as the assumptions regarding the missingness mechanism, are detailed in **Supplementary Materials** (**Section S3**). The imputed dataset was confirmed valid by consistency testing and then used for subsequent analyses. Categorical variables were presented as counts and percentages (n, %). Continuous variables were tested for normality using the Shapiro-Wilk test. Normally distributed variables were expressed as mean ± standard deviation (SD), and non-normally distributed variables were expressed as median (interquartile range, IQR). A two-sided P-value < 0.05 was considered statistically significant.

Progression of T2DKD was defined as the binary outcome, and all baseline characteristics were independent variables. Univariate logistic regression was first conducted for preliminary screening. Variables with P < 0.10 were identified as potential candidate predictors. Least absolute shrinkage and selection operator (LASSO) regression was applied to regularize candidate variables and shrink coefficients. The optimal penalty parameter λ was selected via 10-fold cross-validation. Redundant variables were automatically removed to balance model complexity and predictive performance, and core candidate predictors were identified. Multicollinearity was assessed for variables screened by LASSO regression. A variance inflation factor (VIF) ≥ 10 indicated significant multicollinearity. After excluding severe multicollinearity, the remaining variables were entered into multivariate logistic regression. Independent effects of each variable were evaluated, and independent core predictors of T2DKD progression were finally determined.

The dataset was randomly split into training and test datasets at a ratio of 7:3. Stratified random sampling was employed to preserve outcome distribution, with full reproducibility ensured by explicit random seed setting. Based on the screened core predictors, six predictive models were constructed: logistic regression (LR), random forest (RF), K-nearest neighbors (KNN), support vector machine (SVM), neural network (NN), and extreme gradient boosting (XGBoost). For each machine learning algorithm, hyperparameters were optimized to maximize predictive performance. The neural network architecture, hyperparameter tuning strategy, optimizer, loss function, training epochs, and final selected parameters for all six models are fully described in **Supplementary Materials** (**Section S1**).

Predictive performance was assessed in three domains: (1) discrimination, evaluated using receiver operating characteristic (ROC) curves and area under the curve (AUC); (2) calibration, assessed using calibration curves, the Hosmer-Lemeshow test, and Brier score; and (3) clinical utility, evaluated using decision curve analysis (DCA). A higher AUC (closer to 1) indicated stronger discriminative ability. A P-value > 0.05 for the Hosmer-Lemeshow test suggested good calibration. A Brier score closer to 0 indicated higher predictive accuracy. Clinical net benefit was quantified across different risk thresholds to determine clinical practicality.

To address the inherent “black-box” limitation of ML models, SHapley Additive exPlanations (SHAP) analysis was used for model interpretability. The mean absolute SHAP value was used to quantify the importance of each core feature. These analyses were combined with SHAP feature importance plots and beeswarm plots to examine the regulatory patterns of core features on T2DKD progression risk and population-specific differences. All analyses were performed in both the training datasets and independent test datasets to verify the generalizability of the results.

## 3 Results

### 3.1 Participant enrollment and dataset partitioning

**Figure 1:**
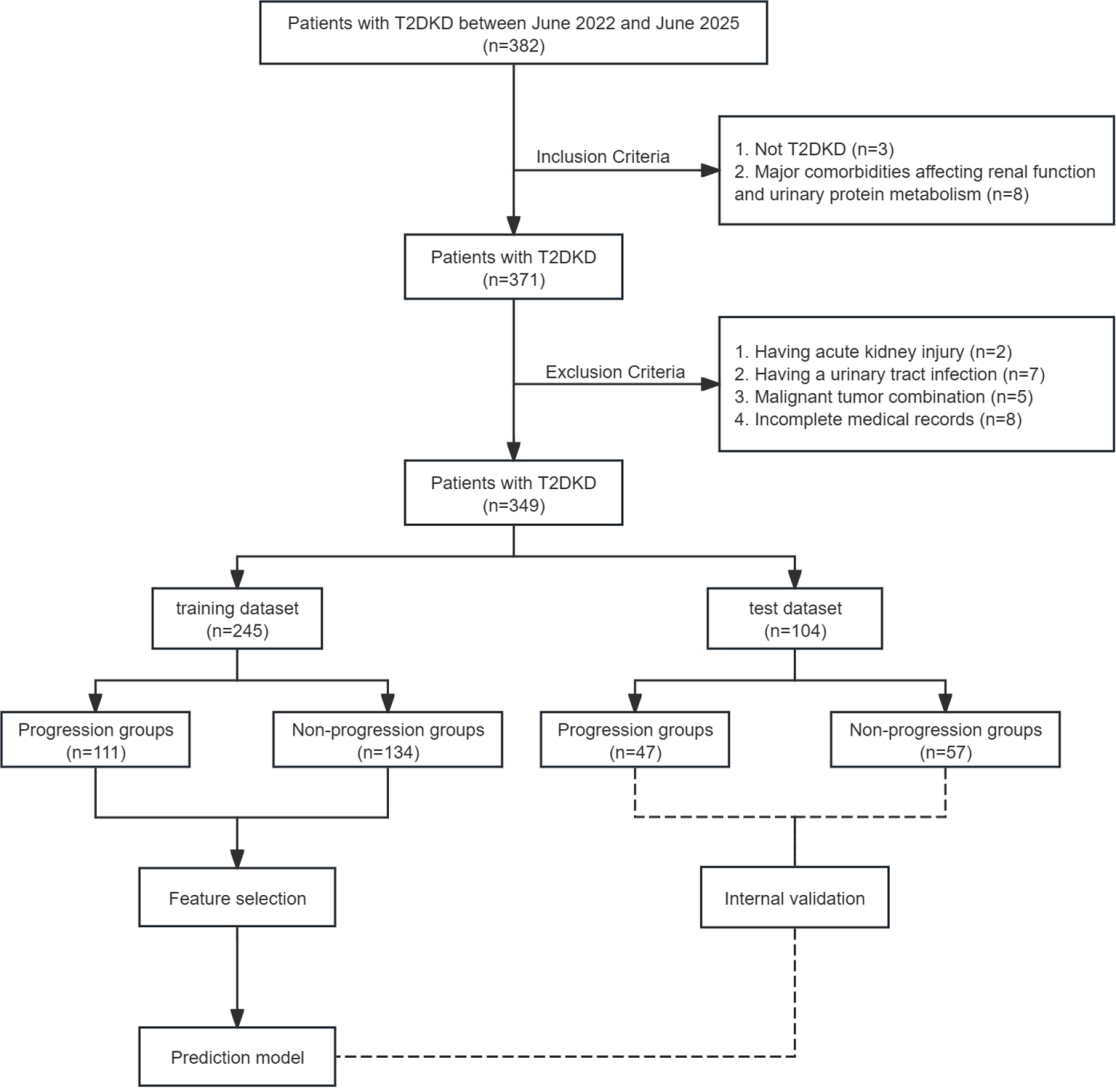
Flowchart of patient selection and model development.

A total of 382 patients with T2DKD were initially enrolled. After screening, 33 ineligible patients were excluded, leaving 349 eligible patients for final analysis (**Figure 1**). The mean follow-up duration was 1.61 ± 0.91 years. Among the included patients, 213 (61.0%) were male and 136 (39.0%) were female. The mean age was 63.18 ± 13.39 years, and the mean diabetes duration was 13.21 ± 7.76 years. During follow-up, 158 (45.3%) patients experienced disease progression and 191 (54.7%) did not. The dataset was randomly divided into training and test datasets at a 7:3 ratio. The training datasets comprised 245 patients (111 in the progression group and 134 in the non-progression group), and the test datasets comprised 104 patients (47 in the progression group and 57 in the non-progression group).

### 3.2 Baseline characteristics of participants

Significant between-group differences were observed in age, prevalence of DPN and MASLD, BMI, FPG, and ALT (all P < 0.05). These baseline differences were considered clinically relevant and were addressed through confounding adjustment in model development.

No significant differences were observed for other variables, including sex, DM duration, HTN duration, smoking status, drinking status, DR, OP, Met use, SGLT2i use, FPG, VD, PTH, and HbA1c, etc. (all P > 0.05). These findings indicated good overall comparability between the two groups. Detailed baseline characteristics are presented in **Table 1**.

**Table 1:** Baseline characteristics between progression and non-progression groups in patients with T2DKD.

Variable	Overall N = 349	Non-progression N = 191	Progression N = 158	P value
Sex				0.900
Female	136 (39.0%)	75 (39.1%)	61 (38.6%)	
Male	213 (61.0%)	116 (60.7%)	97 (61.4%)	
Age (years)				0.009
<60	116 (33.2%)	52 (27.2%)	64 (40.5%)	
≥60	233 (66.8%)	139 (72.8%)	94 (59.5%)	
DM duration (years)				0.062
<10	173 (49.6%)	86 (45.0%)	87 (55.1%)	
≥10	176 (50.4%)	105 (55.0%)	71 (44.9%)	
HTN duration (years)				0.820
<5	168 (48.1%)	93 (48.7%)	75 (47.5%)	
≥5	181 (51.9%)	98 (51.3%)	83 (52.5%)	
Smoking				0.892
No	209 (59.9%)	115 (60.2%)	94 (59.5%)	
Yes	140 (40.1%)	76 (39.8%)	64 (40.5%)	
Drinking				0.357
No	245 (70.2%)	138 (72.3%)	107 (67.7%)	
Yes	104 (29.8%)	53 (27.8%)	51 (32.3%)	
DR				0.745
No	180 (51.6%)	97 (50.8%)	83 (52.5%)	
Yes	169 (48.4%)	94 (49.2%)	75 (47.5%)	
DPN				0.002
No	74 (21.2%)	52 (27.2%)	22 (13.9%)	
Yes	275 (78.8%)	139 (72.8%)	136 (86.1%)	
OP				0.736
No	160 (45.9%)	86 (45.0%)	74 (46.8%)	
Yes	189 (54.2%)	105 (55.0%)	84 (53.2%)	
MASLD				0.001
No	196 (56.2%)	122 (63.9%)	74 (46.8%)	
Yes	153 (43.9%)	69 (36.1%)	84 (53.2%)	
Met use				0.162
No	191 (54.7%)	111 (58.1%)	80 (50.6%)	
Yes	158 (45.3%)	80 (41.9%)	78 (49.4%)	
SGLT2i use				0.170
No	241 (69.1%)	126 (66.0%)	115 (72.8%)	
Yes	108 (31.0%)	65 (34.0%)	43 (27.2%)	
BMI (kg/m²)	24.54 (22.66, 26.99)	24.37 (22.22, 26.62)	24.89 (23.10, 27.62)	0.034
SBP (mmHg)	145.58 ± 19.19	144.52 ± 19.09	146.87 ± 19.28	0.257
DBP (mmHg	78.00 (71.00, 86.00)	78.00 (71.00, 85.50)	78.50 (72.00, 88.00)	0.202
FPG (mmol/L)	7.70 (6.30, 9.70)	7.40 (6.10, 9.40)	7.90 (6.62, 10.38)	0.046
VD (ng/mL)	18.00 (14.00, 23.00)	18.00 (14.00, 25.00)	18.00 (13.00, 23.00)	0.246
PTH (pg/mL)	43.00 (32.00, 61.00)	45.00 (33.00, 64.00)	40.00 (30.00, 56.00)	0.139
HbA1c (%)	8.66 (7.55, 10.41)	8.48 (7.19, 10.29)	8.91 (7.91, 10.66)	0.108
HGB (g/L)	126.42 ± 22.65	127.09 ± 20.41	125.61 ± 25.13	0.552
WBC (×10^9^/L)	6.60 (5.50, 7.80)	6.60 (5.50, 7.70)	6.50 (5.53, 7.90)	0.654
PLT (×10^9^/L)	187.00 (147.00, 232.00)	187.00 (145.50, 232.00)	188.00 (149.00, 231.25)	0.867
ALT (U/L)	19.00 (13.30, 27.60)	17.20 (12.70, 25.40)	20.95 (13.93, 30.65)	0.011
AST (U/L)	18.60 (15.30, 24.00)	18.30 (14.75, 22.55)	19.15 (15.83, 26.40)	0.082
AAR	1.00 (0.80, 1.20)	1.00 (0.80, 1.25)	1.00 (0.80, 1.20)	0.146
ALP (U/L)	80.70 (67.60, 101.10)	79.90 (67.15, 104.10)	81.95 (67.82, 97.25)	0.734
TBIL (μmol/L)	9.30 (7.00, 12.00)	9.20 (7.25, 12.55)	9.30 (6.70, 11.60)	0.612
DBIL (μmol/L)	2.20 (1.50, 3.00)	2.20 (1.60, 3.20)	2.10 (1.50, 2.90)	0.246
ALB (g/L)	39.80 (36.70, 42.80)	39.80 (37.05, 42.65)	39.80 (36.42, 43.18)	0.902
CHOL (mmol/L)	4.16 (3.35, 5.06)	4.15 (3.41, 5.05)	4.25 (3.11, 5.08)	0.693
hs-CRP (mg/L)	2.00 (1.00, 5.00)	2.00 (1.00, 5.00)	2.00 (1.00, 6.00)	0.237
LDL (mmol/L)	2.24 (1.53, 2.90)	2.24 (1.54, 2.85)	2.25 (1.51, 2.91)	0.881
TG (mmol/L)	1.86 (1.24, 2.85)	1.73 (1.29, 2.76)	1.98 (1.16, 2.90)	0.489
HDL (mmol/L)	1.11 (0.93, 1.33)	1.08 (0.95, 1.33)	1.14 (0.92, 1.31)	0.928
BUN (mmol/L)	6.91 (5.48, 9.36)	6.85 (5.62, 8.74)	6.96 (5.17, 10.12)	0.711
UA (μmol/L)	359.30 (292.50, 413.50)	355.30 (293.50, 404.35)	361.15 (291.98, 420.98)	0.680

Variables are expressed as frequency (%), mean ± standard deviation, or median (IQR).

### 3.3 Feature variable selection

**Figure 2:**
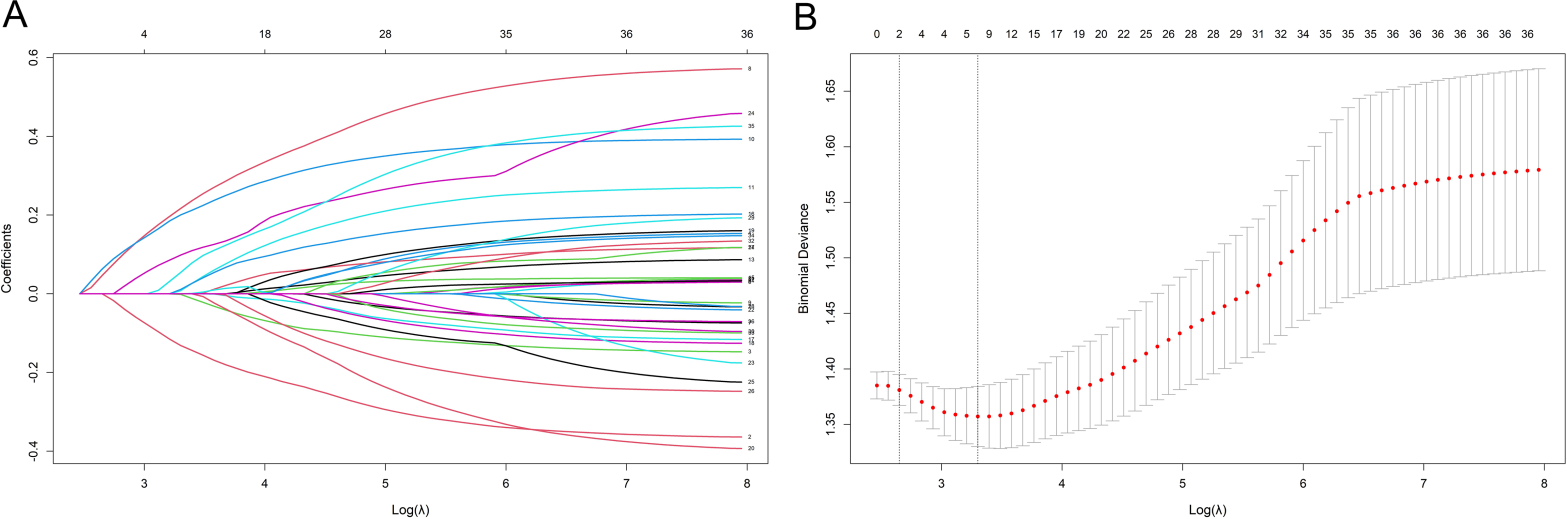
Lasso regression for predictive variable selection in T2DKD progression. **(A)** regularization path of lasso regression coefficients. **(B)** cross-validation curve for selection of optimal regularization parameter λ.

To screen potential predictors of T2DKD progression, we first performed preliminary screening using univariate logistic regression analysis (**Table 2**). With a screening threshold of P < 0.10, a total of 9 potential candidate predictors were identified, namely age, DM duration, DPN, MASLD, BMI, DBP, FPG, ALT, and AST. Subsequently, LASSO regression was used as the core feature selection method to screen potential core predictive variables (**Figure 2**). Diagnostic testing for multicollinearity was performed for the variables screened by LASSO regression (**Table 3**). The results showed that the VIF of all indicators was < 10, indicating no significant multicollinearity.

**Table 2:** Univariable logistic regression analysis for T2DKD progression.

Variables	β coefficient	Standard error	P value	Odds ratio (95%CI)
Sex				
Female				1.00 (Reference)
Male	0.03	0.22	0.900	1.03 (0.67 ~ 1.58)
Age (years)				
<60				1.00 (Reference)
≥60	-0.60	0.23	0.009	0.55 (0.35 ~ 0.86)
DM duration (years)				
<10				1.00 (Reference)
≥10	-0.40	0.22	0.062	0.67 (0.44 ~ 1.02)
HTN duration (years)				
<5				1.00 (Reference)
≥5	0.05	0.22	0.820	1.05 (0.69 ~ 1.60)
Smoking				
No				1.00 (Reference)
Yes	0.03	0.22	0.892	1.03 (0.67 ~ 1.58)
Drinking				
No				1.00 (Reference)
Yes	0.22	0.23	0.357	1.24 (0.78 ~ 1.97)
DR				
No				1.00 (Reference)
Yes	-0.07	0.22	0.745	0.93 (0.61 ~ 1.42)
DPN				
No				1.00 (Reference)
Yes	0.84	0.28	0.003	2.31 (1.33 ~ 4.02)
OP				
No				1.00 (Reference)
Yes	-0.07	0.22	0.736	0.93 (0.61 ~ 1.42)
MASLD				
No				1.00 (Reference)
Yes	0.70	0.22	0.001	2.01 (1.31 ~ 3.09)
Met use				
No				1.00 (Reference)
Yes	0.30	0.22	0.163	1.35 (0.89 ~ 2.07)
No				1.00 (Reference)
Yes	-0.32	0.24	0.171	0.72 (0.46 ~ 1.15)
BMI (kg/m²)	0.06	0.03	0.034	1.06 (1.01 ~ 1.12)
SBP (mmHg)	0.01	0.01	0.256	1.01 (1.00 ~ 1.02)
DBP (mmHg	0.02	0.01	0.093	1.02 (1.00 ~ 1.03)
FPG (mmol/L)	0.07	0.04	0.057	1.08 (1.00 ~ 1.16)
VD (ng/mL)	-0.02	0.01	0.165	0.98 (0.96 ~ 1.01)
PTH (pg/mL)	-0.00	0.00	0.477	1.00 (0.99 ~ 1.00)
HbA1c (%)	0.05	0.05	0.312	1.05 (0.95 ~ 1.16)
HGB (g/L)	-0.00	0.00	0.543	1.00 (0.99 ~ 1.01)
WBC (×10^9^/L)	0.05	0.05	0.321	1.05 (0.95 ~ 1.16)
PLT (×10^9^/L)	0.00	0.00	0.796	1.00 (1.00 ~ 1.00)
ALT (U/L)	0.01	0.01	0.021	1.01 (1.01 ~ 1.02)
AST (U/L)	0.02	0.01	0.018	1.02 (1.01 ~ 1.04)
AAR	-0.40	0.28	0.155	0.67 (0.39 ~ 1.16)
ALP (U/L)	-0.00	0.00	0.677	1.00 (0.99 ~ 1.01)
TBIL (μmol/L)	-0.00	0.02	0.922	1.00 (0.96 ~ 1.04)
DBIL (μmol/L)	-0.03	0.07	0.649	0.97 (0.85 ~ 1.11)
ALB (g/L)	-0.01	0.02	0.787	0.99 (0.95 ~ 1.04)
CHOL (mmol/L)	0.00	0.04	0.911	1.00 (0.92 ~ 1.09)
hs-CRP (mg/L)	0.00	0.01	0.357	1.00 (0.99 ~ 1.02)
LDL (mmol/L)	0.04	0.11	0.697	1.04 (0.85 ~ 1.28)
TG (mmol/L)	0.04	0.04	0.301	1.04 (0.97 ~ 1.12)
HDL (mmol/L)	0.05	0.28	0.857	1.05 (0.61 ~ 1.83)
BUN (mmol/L)	0.03	0.03	0.192	1.03 (0.98 ~ 1.09)
UA (μmol/L)	0.00	0.00	0.767	1.00 (1.00 ~ 1.00)

CI, Confidence Interval. P < 0.1 was considered statistically significant.

**Table 3:** Multivariable logistic regression analysis and multicollinearity testing for T2DKD progression.

Variables	β coefficient	Standard error	P value	Odds ratio (95%CI)	Collinearity statistics	
					Tolerance	VIF
Intercept	-1.43	0.40	<.001	0.24 (0.11 ~ 0.52)	-	-
Age					0.862	1.160
<60				1.00 (Reference)		
≥60	-0.62	0.24	0.011	0.54 (0.34 ~ 0.87)		
DPN					0.980	1.020
No				1.00 (Reference)		
Yes	1.06	0.30	<.001	2.90 (1.61 ~ 5.21)		
MASLD					0.814	1.229
No				1.00 (Reference)		
Yes	0.70	0.23	0.002	2.02 (1.28 ~ 3.18)		
AST	0.02	0.01	0.029	1.02 (1.01 ~ 1.04)	0.374	2.670

P < 0.05 was considered statistically significant. VIF, variance inflation factor. VIF < 10 indicates no significant multicollinearity.

These variables were subsequently entered into a multivariate logistic regression model to analyze the independent associations of each indicator with T2DKD progression (**Table 3**). Age, DPN, MASLD, and AST were identified as independent core predictors of T2DKD progression (all P < 0.05). The feature selection results of LASSO regression were highly consistent with the independent association validation results of multivariate logistic regression, confirming the robustness and core predictive value of these four indicators.

### 3.4 Evaluation and validation of the prediction model

**Figure 3:**
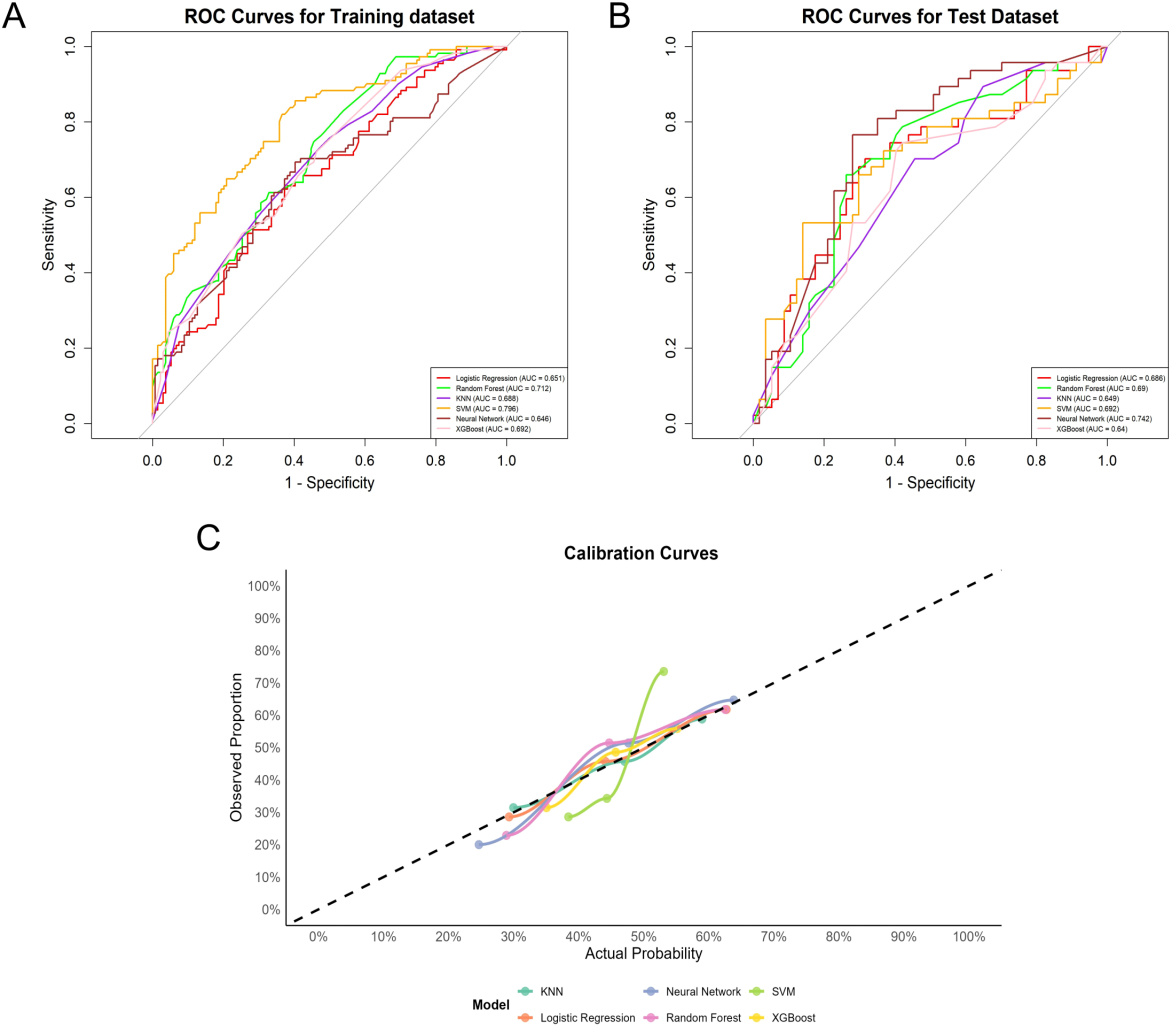
Discrimination and calibration performance of the prediction model for T2DKD progression. **(A)** Receiver operating characteristic (ROC) curve of the model in the training dataset. **(B)** ROC curve of the model in the test dataset. **(C)** Calibration curve of the model in the training dataset.

ROC curve analysis in the training datasets showed that all models demonstrated acceptable discriminative ability (**Figure 3A**). The SVM model achieved the highest AUC of 0.796, followed by the RF model (0.712), XGBoost model (0.692), KNN model (0.688), LR model (0.651), and NN model (0.646). In the test datasets, ROC curve analysis showed consistent discriminative trends with the training datasets, without significant performance degradation (**Figure 3B**). The NN model achieved the highest AUC (0.742), followed by the SVM model (0.692), RF model (0.690), LR model (0.686), KNN model (0.649), and XGBoost model (0.640). These results indicated no significant overfitting and good generalizability across all models. We performed a comprehensive assessment using six core metrics, including accuracy, sensitivity (recall), specificity, positive predictive value (precision), and F1 score (**Table 4**). The test dataset provides the most unbiased measure of real-world generalizability, so we focused our primary analysis on test set performance. The NN model outperformed all other models across every metric.

**Table 4:** Comparison of performance of six machine learning models for T2DKD progression in the training and test datasets.

Model	Dataset	Accuracy	Recall	Specificity	Precision	F1 score	AUC
LR	Training	0.625	0.505	0.724	0.602	0.549	0.651
	Test	0.654	0.532	0.754	0.641	0.581	0.686
RF	Training	0.612	0.667	0.567	0.561	0.609	0.712
	Test	0.664	0.723	0.614	0.607	0.660	0.690
KNN	Training	0.633	0.559	0.694	0.602	0.579	0.688
	Test	0.596	0.468	0.702	0.564	0.512	0.649
SVM	Training	0.722	0.613	0.813	0.731	0.667	0.796
	Test	0.644	0.553	0.719	0.619	0.584	0.692
NN	Training	0.633	0.649	0.619	0.585	0.615	0.646
	Test	0.712	0.809	0.632	0.644	0.717	0.742
XGBoost	Training	0.616	0.279	0.896	0.689	0.397	0.692
	Test	0.587	0.234	0.877	0.611	0.338	0.640

LR, Logistic Regression; RF, Random Forest; KNN, K-Nearest Neighbors; SVM, Support Vector Machine; NN, Neural Network; XGBoost, eXtreme Gradient Boosting.

Calibration performance comparisons are shown in **Figure 3C** and **Table 5**. The NN model demonstrated stable and excellent calibration in both the training and test datasets. HL test P-values were greater than 0.05 in both datasets (0.4724 and 0.2020, respectively), indicating no significant calibration bias. Furthermore, this model achieved the lowest Brier scores (0.2181 in training, 0.2105 in test), suggesting the highest concordance between predicted probabilities and actual T2DKD progression rates. The XGBoost model met calibration criteria only in the test datasets (P = 0.0756), with marginal bias in the training datasets. The LR and KNN models showed acceptable calibration stability but inferior accuracy compared with the NN model. The RF model exhibited significant calibration bias in both datasets, whereas the SVM model demonstrated insufficient calibration generalizability.

**Table 5:** Calibration metrics of six machine learning models for T2DKD progression in the training and test datasets.

Model	Dataset	Brier score	HL-χ²	DF	HL P
LR	Training	0.228	6.229	8	0.622
	Test	0.225	8.067	8	0.427
RF	Training	0.225	22.032	8	0.005
	Test	0.236	20.269	8	0.009
KNN	Training	0.222	4.358	7	0.738
	Test	0.233	3.429	6	0.753
SVM	Training	0.242	9.899	8	0.272
	Test	0.229	17.619	8	0.024
NN	Training	0.218	7.611	8	0.472
	Test	0.211	10.995	8	0.202
XGBoost	Training	0.226	14.648	5	0.012
	Test	0.236	11.445	6	0.076

Brier Score reflects the calibration accuracy of the model; HL-χ², Hosmer-Lemeshow chi-square statistic; DF, degrees of freedom. HL P, Hosmer–Lemeshow test P value; P > 0.05 indicates good calibration of the model.

**Figure 4:**
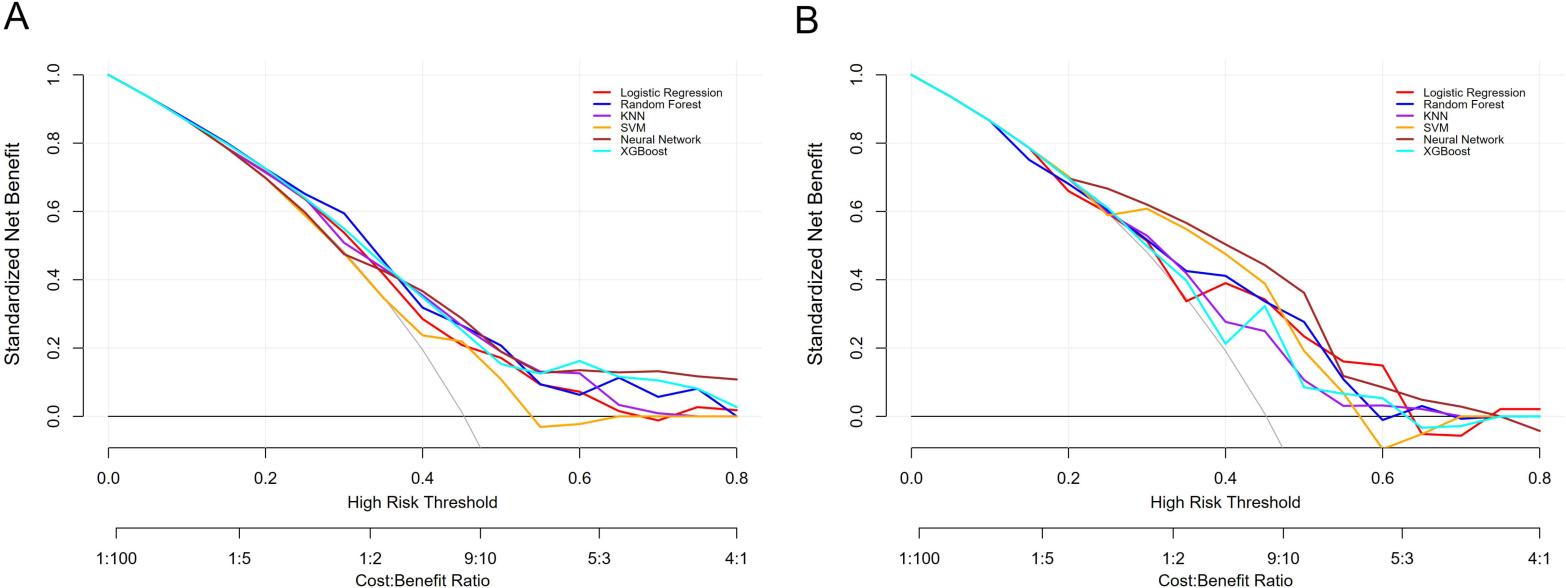
Decision curve analysis (DCA) of the prediction model for T2DKD progression. **(A)** DCA curve in the training dataset. **(B)** DCA curve in the test dataset.

Decision curve analysis results are shown in **Figure 4**. All six models demonstrated positive net benefits above the reference lines across clinically reasonable threshold ranges, indicating clinical utility for T2DKD progression risk stratification. The NN model consistently achieved the highest net benefit across the full range of threshold probabilities. Particularly within the 0.2–0.6 threshold range commonly used in clinical practice, this model maintained superior net benefit, demonstrating optimal clinical applicability.

Based on comprehensive evaluation across discrimination, calibration, and clinical utility dimensions, the NN model was identified as the optimal predictive model for T2DKD progression.

### 3.5 SHAP interpretation for the neural network model

**Figure 5:**
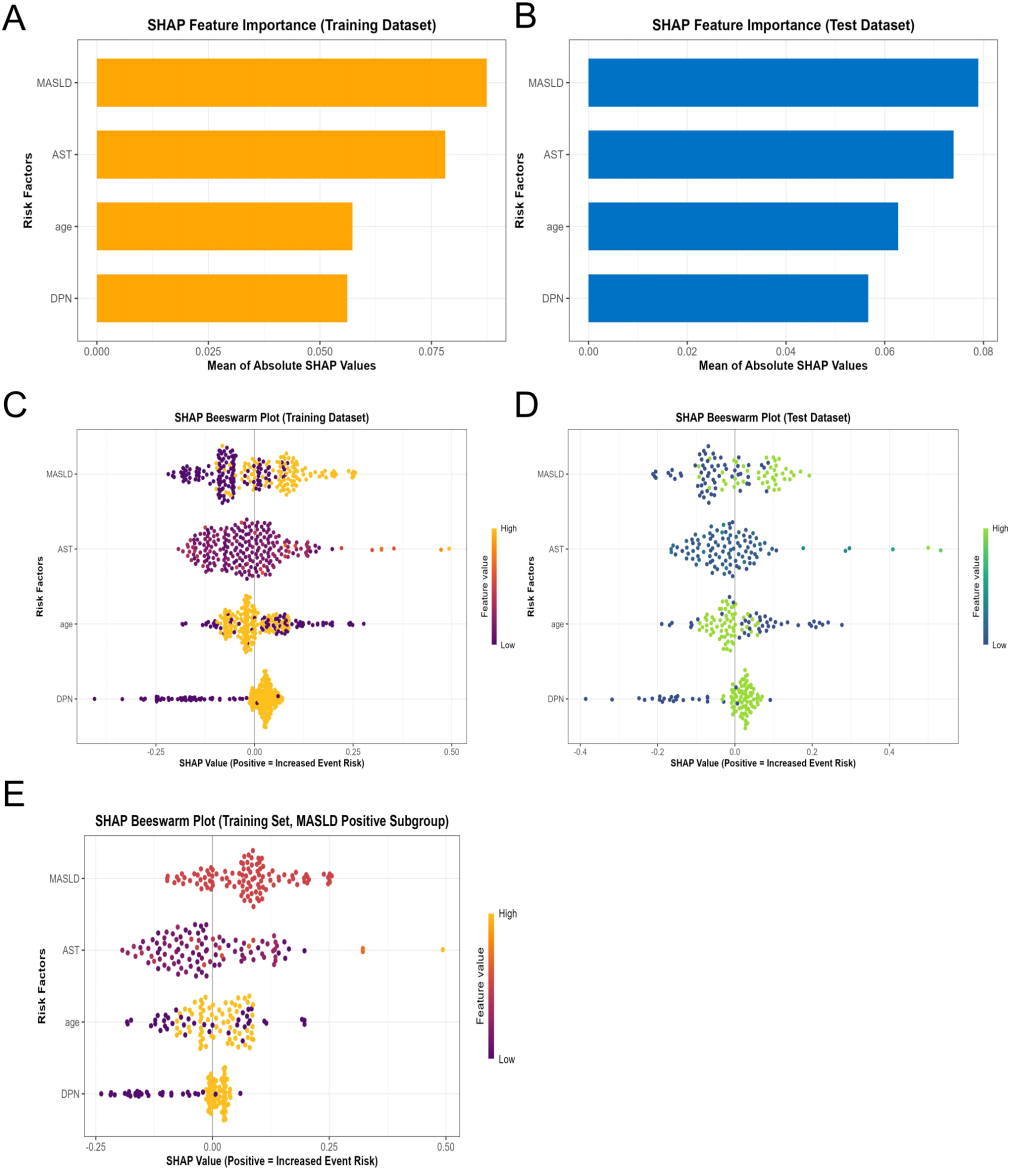
SHAP analysis of the neural network model for predicting T2DKD progression. **(A)** SHAP feature importance bar plot in the training dataset. **(B)** SHAP feature importance bar plot in the test dataset. **(C)** SHAP beeswarm plot in the training dataset. **(D)** SHAP beeswarm plot in the test dataset. **(E)** SHAP beeswarm plot in the training dataset for the MASLD positive subgroup.

We ranked the four risk factors by their contribution to model prediction in the training dataset (**Figure 5A**). MASLD showed the highest contribution (mean SHAP value = 0.0874), followed by AST (0.0781), age (0.0573), and DPN (0.0561). The same ranking appeared in the test dataset, with MASLD contributing 0.0790, AST 0.0740, age 0.0627, and DPN 0.0567. MASLD and AST emerged as the two core predictors (**Figure 5B**). Both showed mean absolute SHAP values above 0.07, contributing significantly more to outcome prediction than DPN or age.

The feature importance rankings showed strong consistency between the training and test datasets (Pearson r = 0.976). This suggests that the model captured robust feature-outcome associations from the training data that generalized well to unseen samples. Stability validation further confirmed these findings. Correlation coefficients ranged from 0.843 to 0.981 (mean = 0.935 ± 0.047; SD = 0.0465). This low variability (SD < 0.05) indicates high stability of the SHAP analysis.

SHAP beeswarm plots revealed positive shifts in SHAP values for MASLD, AST, and DPN when these indicators were elevated (**Figures 5C, D**). This indicates that the model assigned greater predictive weight to elevated levels of these biomarkers. In contrast, SHAP values for age fluctuated around zero, indicating a limited independent predictive contribution relative to the other features.

We next examined the MASLD-positive subgroup (**Figure 5E**). In patients with MASLD alone, SHAP values for MASLD itself fluctuated around zero, suggesting that its isolated presence did not substantially alter the model’s predicted probability. In contrast, AST and DPN retained their positive predictive contributions in this subgroup. These findings suggest that the model relied on the joint presence of MASLD with AST and DPN to refine risk estimation, rather than on MASLD alone.

Overall, the NN model leveraged concurrent variations in markers of liver injury, hepatic steatosis, and metabolic dysfunction to inform its predictions. These predictive patterns resonate with established clinical pathophysiology. Moreover, model interpretability remained stable across both datasets. The distinct contribution profile observed in the MASLD-positive subgroup offers a predictive basis for refining risk stratification and generates hypotheses for subsequent mechanistic inquiry.

## 4 Discussion

This study addressed the need for predicting disease progression risk in patients with established type 2 diabetic kidney disease (T2DKD). We developed a rigorous variable screening procedure to identify robust predictors from 36 baseline characteristics. Four independent core predictors emerged: age, DPN, MASLD, and AST. Using these indicators, we built six ML prediction models. Multi-dimensional assessment verified that the NN model performed optimally for predicting T2DKD progression risk.

MASLD ranked as the leading predictive feature in this study. This finding aligns closely with conclusions from multiple previous clinical studies. This is consistent with the understanding that MASLD is not merely a hepatic disorder, but also a key risk factor associated with diabetic renal injury (20–22). The two conditions share insulin resistance as their core pathological basis. Hepatic steatosis induced by MASLD exacerbates systemic insulin resistance, which in turn triggers glomerular hyperfiltration and hyperperfusion, and disrupts the integrity of the glomerular filtration barrier (23). Meanwhile, MASLD activates local hepatic and systemic chronic low-grade inflammation, which stimulates intrarenal inflammatory pathways via circulating pro-inflammatory cytokines, leading to podocyte apoptosis and glomerular mesangial matrix proliferation (24–26). In addition, profibrotic factors secreted by the liver further drive renal interstitial fibrosis and accelerate the progression of proteinuria (27, 28). SHAP analysis in this study revealed a consistent pattern of joint contribution among MASLD, AST, and DPN. This pattern reveals that the model capitalizes on the joint presence of these features to refine risk stratification, a predictive dynamic that resonates with the multi-pathway pathophysiology posited in experimental literature. These findings are hypothesis-generating. They are compatible with the critical role of the liver-kidney axis in diabetic complications, though they do not establish this role. Whether targeted intervention for hepatic steatosis delays T2DKD progression requires prospective interventional studies.

AST was identified as an independent core predictor of T2DKD progression, expanding the traditional view of AST as a mere liver disease marker. AST is mainly located in hepatocyte mitochondria, and elevated serum AST levels essentially reflect mitochondrial structural and functional impairment (29). Systemic oxidative stress caused by mitochondrial dysfunction directly damages the cytoskeleton of glomerular podocytes and the structure of the glomerular basement membrane, increasing basement membrane permeability. It also impairs the protein reabsorption function of renal tubular epithelial cells, ultimately leading to increased urinary protein excretion (30, 31). Elevated AST often coexists with MASLD, and the two factors frequently co-occur and are jointly associated with lipid metabolic disorders and systemic inflammation (20). This pattern is consistent with the liver-kidney axis hypothesis and may reflect an underlying pathophysiological link that increases T2DKD progression risk. These results suggest that AST, as a convenient serological indicator, may serve as a proxy indicator for the potential renal injury risk in patients with T2DKD.

DPN, one of the most common microvascular complications of diabetes, was identified as a core predictive feature of T2DKD progression in this study. This finding is consistent with the recognized clustering of diabetic microvascular complications. Both conditions share core pathological mechanisms mediated by hyperglycemia, including oxidative stress, microvascular endothelial dysfunction, and chronic inflammation (32). The occurrence of DPN indicates that systemic microvascular injury has reached a significant stage. Pro-inflammatory factors released from damaged nerves trigger systemic low-grade inflammation, aggravating intrarenal inflammation and fibrosis via circulating effects (33). Autonomic dysfunction caused by DPN further activates the renin-angiotensin-aldosterone system (RAAS). This leads to renal hemodynamic disorders, aggravated glomerular hypertension, and destruction of the filtration barrier (34). In addition, neurotrophic factor deficiency not only mediates peripheral nerve injury but also impairs the repair capacity of renal cells, accelerating T2DKD progression (35).

In this study, T2DKD patients aged <60 years showed significantly higher progression risk than those aged ≥60 years, which deviates from conventional clinical understanding. The discrepancy stems not only from study design biases but also from unique pathophysiological and clinical management characteristics of young and middle-aged patients. In terms of study design, most elderly enrolled patients had stable DM duration, while young and middle-aged patients were mostly in active disease stages with poor glycemic and metabolic control, leading to selection bias. Meanwhile, elderly high-risk patients were frequently excluded from analysis due to death from severe cardiovascular or cerebrovascular comorbidities, or loss to follow-up. This resulted in survival bias. Pathophysiologically, young and middle-aged individuals may present more severe early-onset metabolic syndrome and insulin resistance (36). They experience larger glycemic fluctuations, which overactivate renal oxidative stress and nuclear factor-κB inflammatory pathways. Consequently, glomerular hyperfiltration and hyperperfusion are aggravated, and the glomerular filtration barrier becomes disrupted (37). Young and middle-aged patients carrying DKD-susceptible genes (e.g., ACE, APOL1) have significantly increased renal injury susceptibility (38, 39). In this age group, epigenetic regulation is easily disturbed by metabolic disorders, leading to abnormal expression of renal fibrosis-related genes (e.g., TGF-β1, CTGF) and driving irreversible renal injury (40). Additionally, young and middle-aged patients often pay insufficient attention to chronic diabetes management. Early microalbuminuria is asymptomatic. Consequently, renal injury typically remains undetected until the stage of massive albuminuria. Clinical practice should therefore strengthen screening for young and middle-aged patients with diabetic kidney disease, rather than focusing exclusively on elderly patients. This finding remains controversial and requires further validation in larger prospective cohorts.

The NN model developed in this study demonstrated superior overall performance compared with other models. Most previous DKD risk prediction studies used traditional ML methods (e.g., LR, RF), which have inherent limitations in capturing complex non-linear relationships. In contrast, the NN model leverages its multi-layer structure and non-linear activation functions to effectively identify the complex interactions among Age, DPN, MASLD, and AST, achieving higher predictive accuracy. Meanwhile, the model maintained favorable calibration performance and clinical net benefit in both the training and test datasets, confirming its excellent generalizability and clinical practicability.

This study has several notable strengths. First, a rigorous feature selection framework was established, and the robustness of core features was verified via stepwise screening and cross-validation (univariate analysis, LASSO regression, multicollinearity diagnosis, multivariable regression). Furthermore, the included indicators overcome the limitation of most existing studies that focus solely on renal-specific parameters. We constructed a predictive indicator system that takes multi-system interactions into account, which is more consistent with the multifactorial pathogenesis of T2DKD progression. Although MASLD diagnosis conventionally requires abdominal ultrasonography, this examination is already a standard component of annual complication screening for T2DM patients in real-world Chinese clinical practice. Consequently, MASLD status is frequently documented in the electronic medical record at the time of T2DKD diagnosis, incurring no marginal cost for risk prediction. Second, the reliability and stability of the predictive model were ensured through multi-model comparison and three-dimensional evaluation covering discrimination, calibration, and clinical benefit. Third, we performed SHAP interpretation and stability validation for the best-performing NN model, as well as SHAP beeswarm plot analysis specifically for the MASLD-positive subgroup. This enhances the transparency of the NN model by quantifying how each feature influences prediction outcomes. The observed joint contribution patterns in the MASLD-positive subgroup resonate with established pathophysiological hypotheses, affording a predictive vantage point from which to formulate biological questions that ultimately require experimental resolution. Finally, the NN model developed herein provides an efficient and scalable tool for routine clinical practice and community health management. It holds considerable potential for broad clinical application.

This study also has several limitations. First, the highest AUC among all models, including the NN, was 0.742 in the test dataset. This indicates that discriminative ability for T2DKD progression remains suboptimal and warrants improvement. This limitation may be attributed to the fact that DKD is a complex disease caused by multiple factors, and not all potential variables were included in the baseline data of this study. Second, this was a single-center retrospective cohort study, which can only reveal the correlation between variables but cannot confirm causal relationships. Finally, only internal validation of the model was completed in this study, and independent external cohort validation was not performed. Therefore, the generalizability of the model still needs further confirmation. Based on these limitations, future studies should expand the sample size, conduct multi-center prospective cohort research, and incorporate additional potential influencing factors. External validation and model optimization are essential next steps. Furthermore, in-depth exploration of specific molecular mechanisms through which core predictors mediate T2DKD progression is warranted. Such research would provide novel theoretical foundations for early intervention strategies.

## 5 Conclusion

This study identified four core risk factors for T2DKD progression: MASLD, AST, DPN, and age. The NN prediction model constructed using these routine clinical indicators demonstrates favorable discrimination, calibration, and clinical interpretability. It effectively predicts proteinuria progression risk in T2DKD patients. This model provides a cost-effective tool for screening high-risk populations with T2DKD progression, which is suitable for clinical practice and community health services, and offers a reference for early intervention and prognosis improvement of T2DKD.

## Data Availability

The raw data supporting the conclusions of this article will be made available by the authors, without undue reservation.
