# Supplementary material for: Development and validation of an explainable neural network model for predicting progression in type 2 diabetic kidney disease
Source: Front Endocrinol (Lausanne). 2026 May 28;17:1858808. doi: 10.3389/fendo.2026.1858808 (PMC13253395; doi:10.3389/fendo.2026.1858808)
Supplement: Supplementary file 1 [file DataSheet1.doc]

**Supplementary Materials**

**S1. Supplementary Methods: Model Development and Hyperparameter Tuning**

**S1.1 Neural Network Architecture and Training Protocol**

The neural network (NN) model was constructed using the neuralnet package (version 1.44.2) in R. The architecture was determined through a systematic grid search over hidden layer configurations, with the following specifications for the final optimal model:

**Architecture Details:**

- Input layer: 4 neurons (corresponding to the 4 selected features: age, DPN, MASLD, AST)
- Hidden layers: 1 hidden layer with 5 neurons (configuration: hidden = c(5))
- Output layer: 1 neuron with sigmoid activation for binary classification
- Activation function: Logistic sigmoid function (default in neuralnet)
- Linear output: linear.output = FALSE (appropriate for binary classification)
- Weight initialization range: rang = 0.1 (uniform distribution, ±0.1)

**Training Hyperparameters:**

- Optimizer: Resilient Backpropagation (Rprop+, default in neuralnet package)
- Loss function: Sum of squared errors (SSE)
- Maximum training iterations (epochs): maxit = 1000
- Convergence threshold: Algorithm-specific stopping criteria based on partial derivatives (default neuralnet settings)
- Error function: Sum of squared errors (SSE)

**Hyperparameter Tuning Strategy:**

A comprehensive grid search was performed over the following hidden layer configurations to identify the optimal architecture: hidden = c(2), c(3), c(4), c(5), c(2, 1), c(2, 2). The optimal configuration (hidden = c(5)) was selected based on the highest Area Under the Receiver Operating Characteristic Curve (AUC) evaluated on the independent test set. No dropout regularization was applied, as the neuralnet package does not natively support dropout layers. Overfitting was mitigated through: (1) Feature selection (LASSO + univariate screening) reducing dimensionality to 4 variables; (2) Early stopping via maxit limitation; (3) Weight decay equivalent through the decay parameter (set to 0.01 in the final SHAP analysis model).

**S1.2 Random Forest (RF) Hyperparameter Tuning**

The Random Forest model was implemented using the randomForest package (version 4.7-1.1) and caret package (version 6.0-94) for systematic hyperparameter optimization.

**Table S1. Random Forest Hyperparameter Search Space and Final Values**

| **Parameter** | **Search Range** | **Final Value** | **Description** |
| --- | --- | --- | --- |
| mtry | {1, 2} | 2 | Number of variables randomly sampled at each split |
| ntree | {50, 75, 100, 125, 150, 175, 200} | 150 | Number of trees in the forest |
| maxnodes | 8 | 8 | Maximum number of terminal nodes per tree |
| min.node.size | 5 | 5 | Minimum size of terminal nodes |
| classwt | c(Yes=1, No=1) | c(Yes=1, No=1) | Class weights |

Tuning Strategy: (1) Grid search via caret::train() with 10-fold cross-validation, repeated 3 times (repeats = 3); (2) Optimization metric: ROC-AUC (metric = "ROC"); (3) OOB error validation: Additional validation of ntree using out-of-bag error rates to confirm optimal tree count.

**S1.3 XGBoost Hyperparameter Tuning**

The XGBoost model was implemented using the xgboost package (version 1.6.0.1).

**Table S2. XGBoost Hyperparameter Search Space and Final Values**

| **Parameter** | **Search Range** | **Final Value** | **Description** |
| --- | --- | --- | --- |
| max_depth | {2, 3, 4, 5} | 4 | Maximum depth of trees |
| eta (learning rate) | {0.01, 0.1, 0.2} | 0.1 | Learning rate/shrinkage |
| nrounds | {50, 100, 150} | 150 | Number of boosting rounds |
| objective | "binary:logistic" | "binary:logistic" | Binary classification objective |

Tuning Strategy: Exhaustive grid search over all 36 combinations (4 × 3 × 3) of the above parameters. The optimal combination was selected based on the highest test set AUC.

**S1.4 Support Vector Machine (SVM) Hyperparameter Tuning**

The SVM model was implemented using the e1071 package (version 1.7-13).

**Table S3. SVM Hyperparameter Search Space and Final Values**

| **Parameter** | **Search Range** | **Final Value** | **Description** |
| --- | --- | --- | --- |
| kernel | "radial" | "radial" | Radial basis function (RBF) kernel |
| cost (C) | 10^(-1:3) = {0.1, 1, 10, 100, 1000} | 10 | Penalty parameter |
| gamma | 10^(-3:1) = {0.001, 0.01, 0.1, 1, 10} | 0.01 | Kernel coefficient |

Tuning Strategy: 10-fold cross-validation via tune.svm() with tune.control(sampling = "cross", cross = 10).

**S1.5 k-Nearest Neighbors (k-NN) Hyperparameter Tuning**

The k-NN model was implemented using the kknn package (version 1.3.1).

**Table S4. k-NN Hyperparameter Search Space and Final Values**

| **Parameter** | **Search Range** | **Final Value** | **Description** |
| --- | --- | --- | --- |
| k (neighbors) | {15, 17, 19, ..., 49} | 35 | Number of neighbors (step = 2) |
| kernel | "rectangular" | "rectangular" | Unweighted rectangular kernel |

Tuning Strategy: 5-fold cross-validation AUC was used as the primary selection criterion to prevent test set information leakage. The kernel was fixed to "rectangular" (unweighted) to minimize overfitting.

**S1.6 Logistic Regression**

Standard multivariable logistic regression was fitted using glm() with family = binomial(link = "logit"). No regularization was applied. The model included all 4 selected features (age, DPN, MASLD, AST) simultaneously.

**S2. Software and Reproducibility**

**S2.1 Software Environment**

All analyses were conducted in R version 4.3.1 (or later compatible version) running on Windows 10/11 operating system. The following R packages and versions were used:

**Table S5. R Packages and Versions Used in This Study**

| **Package** | **Version** | **Purpose** |
| --- | --- | --- |
| neuralnet | 1.44.2 | Neural network modeling |
| NeuralNetTools | 1.5.3 | Neural network visualization |
| randomForest | 4.7-1.1 | Random forest modeling |
| caret | 6.0-94 | Machine learning framework, cross-validation |
| xgboost | 1.6.0.1 | Gradient boosting |
| kknn | 1.3.1 | k-nearest neighbors |
| e1071 | 1.7-13 | Support vector machine |
| glmnet | 4.1-7 | LASSO feature selection |
| missForest | 1.5 | Missing data imputation |
| mice | 3.16.0 | Missing data analysis |
| pROC | 1.18.4 | ROC curve analysis |
| rmda | 1.6 | Decision curve analysis |
| kernelshap | 0.3.5 | SHAP value computation |
| shapviz | 0.9.2 | SHAP visualization |
| ggplot2 | 3.4.4 | Data visualization |
| tableone | 0.13.2 | Baseline characteristics table |

**S2.2 Random Seeds and Reproducibility**

The following random seeds were explicitly set at critical steps to ensure full reproducibility:

**Table S6. Random Seeds Used for Reproducibility**

| **Analysis Step** | **Random Seed** | **Function** |
| --- | --- | --- |
| Missing data imputation (missForest) | set.seed(123) | missForest() |
| Data splitting (train/test) | set.seed(52) | caret::createDataPartition() |
| Data standardization | set.seed(111) | scale() |
| k-NN cross-validation | set.seed(123) | createFolds() |
| RF hyperparameter tuning | set.seed(123) | caret::train() |
| SVM tuning | set.seed(11) | tune.svm() |
| SHAP analysis | set.seed(123) | Background sampling |
| SHAP stability validation | set.seed(123) | Repeated background sampling |

Data splitting protocol: Stratified random split using caret::createDataPartition(y = data$Result, p = 0.7, list = FALSE), ensuring 70% training and 30% testing with preserved outcome distribution.

**S3. Missing Data Handling**

**S3.1 Proportion and Pattern of Missingness**

Missing data were present in 3 continuous variables out of a total of 26 variables (1 outcome + 25 predictors). The missing data pattern was assessed prior to imputation:

**Table S7. Missing Data Proportion by Variable**

| **Variable** | **Type** | **Missing Percentage** | **Missing Pattern** |
| --- | --- | --- | --- |
| VD (Vitamin D) | Continuous | 2.58% | Always co-occurs with PTH (never isolated) |
| PTH (Parathyroid Hormone) | Continuous | 2.58% | Always co-occurs with VD (never isolated) |
| hs-CRP (High-sensitivity CRP) | Continuous | 5.44% | Always isolated (never co-occurs with VD/PTH) |
| Complete cases | — | 91.98% | No missing data in any variable |
| Note: Based on the code, these 3 variables were identified as having missing values via sum(is.na()) checks. | | | |

**Missingness pattern summary:** Among 349 total participants, 321 (91.98%) had complete data for all variables. The remaining 28 participants (8.02%) exhibited missing data in 2 distinct patterns: (1) simultaneous VD+PTH missing in 9 cases (2.58%), with no isolated VD or PTH missing; and (2) isolated hs-CRP missing in 19 cases (5.44%), with no co-occurrence with VD or PTH. No participants had all three variables missing, nor did any have PTH+hs-CRP or VD+hs-CRP missing without the third variable.

**Missingness mechanism assumption:** We assumed the data were Missing At Random (MAR). The co-occurrence pattern of VD and PTH (always missing together) suggests these laboratory tests were likely ordered as a bundled metabolic bone panel, supporting a MAR mechanism where missingness depends on observed clinical characteristics (e.g., physician ordering patterns based on patient comorbidities) rather than the unobserved values themselves. The isolated hs-CRP missingness may reflect selective testing in specific clinical scenarios. The missForest algorithm is robust to MAR assumptions as it leverages relationships between all observed variables for imputation.

**S3.2 Imputation Methodology**

Method: Random Forest imputation using the missForest package

**Algorithm parameters:**

- Iterations: Default (algorithm-converged)
- Variable-wise error convergence: Default stopping criteria
- Parallel processing: Not enabled (parallelize = "no", default)

**Post-imputation processing:**

- VD, PTH, and hs-CRP were rounded to integers after imputation to maintain clinical interpretability (round() function)
- Imputation quality was visually validated by comparing original vs. imputed distributions via histogram overlays

Validation of imputation quality: The distributions of imputed values were compared with observed distributions using histograms (Supplementary Figure S1). No significant distributional distortions were observed, supporting the validity of the imputation.

| **A** | **B** |
| --- | --- |
| 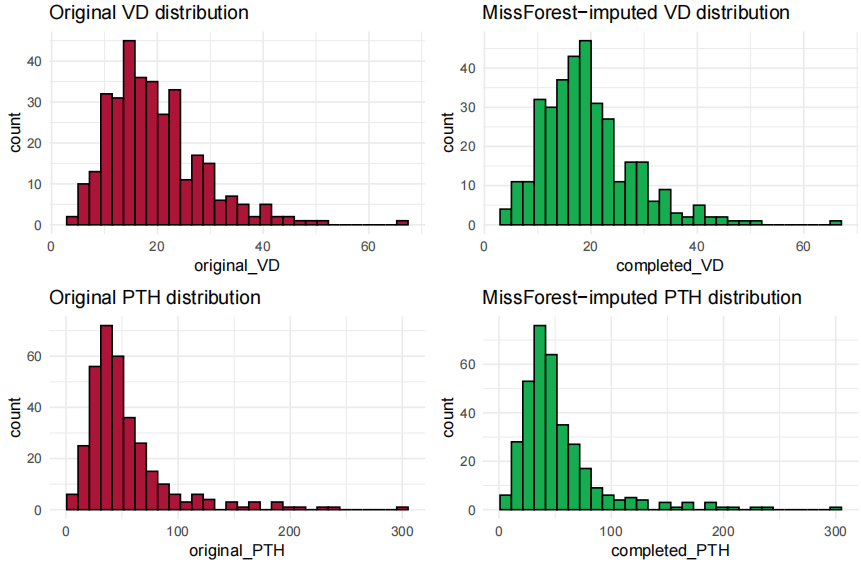 | 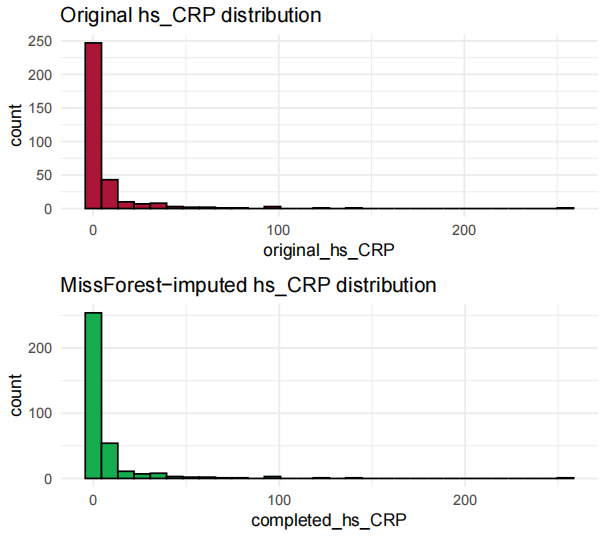 |

**Figure S1. Validation of missForest imputation by distribution comparison.**

**S4. Feature Selection and Data Preprocessing**

**S4.1 Feature Selection Strategy**

A two-stage feature selection approach was employed:

**Stage 1: Univariate screening + Multivariable logistic regression**

- Univariate logistic regression for each candidate variable
- Multivariable logistic regression with backward selection (threshold: P < 0.05)
- Implemented via autoReg package

**Stage 2: LASSO (Least Absolute Shrinkage and Selection Operator)**

- glmnet package with alpha = 1 (L1 regularization)
- 10-fold cross-validation to select optimal lambda (cv.glmnet)
- Optimal lambda: lambda.min (or manually specified lambda = 0.05343 for feature retention)

**Final selected features (4 variables): age, DPN, MASLD, AST**

**S4.2 Data Standardization**

Continuous variables (AST) were standardized using z-score normalization (scale() function) before neural network and k-NN model training:

*xscaled = (x - μ) / σ*

Categorical variables (age, DPN, MASLD) were retained as binary dummy variables (0/1).

**S5. Model Evaluation and Validation**

**S5.1 Performance Metrics**

Models were evaluated using the following metrics:

- Discrimination: Area Under the ROC Curve (AUC) with 95% confidence intervals
- Calibration: Brier score, Hosmer-Lemeshow goodness-of-fit test (dynamic grouping: g = min(10, max(3, floor(min_class/2))))
- Clinical utility: Decision Curve Analysis (DCA) with threshold probabilities from 0 to 0.8 (step = 0.05), 10 bootstraps
- Optimal threshold: Youden index (sensitivity + specificity - 1)

**S5.2 SHAP (SHapley Additive exPlanations) Analysis**

**Implementation: kernelshap package with exact Kernel SHAP computation.**

**Parameters:**

- Background dataset: Random sample of 50-100 observations from training set
- Prediction wrapper: Custom function extracting "Event" class probability from nnet model
- Parallel processing: Disabled (n_cores = 1) for reproducibility
- Stability validation: 10 repeated computations with resampled background sets; standard deviation of feature importance correlations < 0.03 considered stable

**S6. Data Availability and Code Availability**

The R code used for all analyses is available upon reasonable request to the corresponding author. The dataset cannot be made publicly available due to patient privacy restrictions but may be accessed through formal data sharing agreements with the corresponding author.

*Corresponding Author Contact Information:
E-mail: 2001090207@163.com*
